# Prognostic Value of the Systemic Inflammatory Response Index in Patients Undergoing Radical Cystectomy for Bladder Cancer: A Population-Based Study

**DOI:** 10.3389/fonc.2021.722151

**Published:** 2021-08-16

**Authors:** Jinliang Ni, Keyi Wang, Houliang Zhang, Jinbo Xie, Jun Xie, Changxiu Tian, Yifan Zhang, Weiyi Li, Bin Su, Chaozhao Liang, Xinran Song, Bo Peng

**Affiliations:** ^1^Department of Urology, Shanghai Tenth People’s Hospital, Tongi University, Shanghai, China; ^2^Shanghai Clinical College, Anhui Medical University, Hefei, China; ^3^Department of Urology, Shanghai Tenth People’s Hospital, School of Medicine, Tongji University, Shanghai, China; ^4^Department of Blood Transfusion, Shanghai Tenth People’s Hospital, School of Medicine, Tongji University, Shanghai, China; ^5^Department of Urology, First Affiliated Hospital of Anhui Medical University, Anhui Medical University, Hefei, China; ^6^Department of Medical Ultrasound, Shanghai Tenth People’s Hospital, Ultrasound Research and Education Institute, Tongji University Cancer Center, Shanghai Engineering Research Center of Ultrasound Diagnosis and Treatment, Tongji University School of Medicine, Shanghai, China

**Keywords:** systemic inflammation response index, bladder cancer, radical cystectomy, nomogram, prognosis

## Abstract

**Purpose:**

The aim of this study was to evaluate the prognostic significance of the systemic inflammatory response index (SIRI) in patients with bladder cancer (BCa) treated with radical cystectomy (RC) and develop a survival predictive model through establishing a nomogram.

**Materials and Methods:**

A total of 203 BCa patients who underwent RC were included in this study. The relationship between the SIRI and overall survival (OS), disease-free survival (DFS), and clinicopathological features were evaluated. Cox regression analysis was performed to investigate the effect of the factors on the OS and DFS. The results were applied in the establishment of a nomogram. Receiver operating characteristic (ROC) curves, decision curve analysis (DCA) curves, and calibration curves were performed to assess the predictive performance and accuracy of the nomogram, respectively.

**Results:**

According to the classification of the SIRI, 81 patients (39.9%) were assigned to SIRI grade 1, 94 patients (46.3%) to SIRI grade 2, and the remaining 28 patients (13.8%) to SIRI grade 3. Multivariate Cox regression revealed that a higher SIRI grade was significantly associated with a poor prognosis and served as an independent prognostic factor for the OS [Grade 2 *vs* Grade 1, odds ratio = 2.54, 95% confidence interval (CI),1.39–4.64, P = 0.002; Grade 3 *vs* Grade 1, odds ratio = 4.79, 95%CI: 2.41–9.50, P < 0.001] and DFS [Grade 2 *vs* Grade 1, odds ratio = 2.19, 95% CI, 1.12–4.31, P = 0.023; Grade 3 *vs* Grade 2, odds ratio = 3.36, 95%CI, 1.53–7.35, P = 0.002]. The ROC and DCA analysis indicated that the nomogram based on the SIRI contained a better predictive performance compared with the TNM stage (AUC = 0.750 and 0.791; all P < 0.05). The ROC analysis showed that nomograms can better predict the 3- and 5-year OS and DFS. The calibration curves exhibited a significant agreement between the nomogram and the actual observation.

**Conclusion:**

SIRI as a novel independent prognostic index and potential prognostic biomarker can effectively improve the traditional clinicopathological analysis and optimize individualized clinical treatments for BCa patients after RC.

## Introduction

Bladder cancer (BCa) as the 10^th^ most prevalent cancer worldwide with high morbidity and mortality rates carries a large societal burden (1). There are two main classifications of BCa: more than 75% of patients are diagnosed with non-muscle-invasive BCa (NMIBC), whereas muscle-invasive BCa (MIBC) accounts for approximately 25% of BCa patients (2). NMIBC comprises stage Ta, T1, and carcinoma *in situ* (CIS), which contains the infiltration of the cancer cells into the mucosa and submucosa of the bladder (3). In contrast, the cancer cells that invade the muscle layer are referred to as MIBC and have a higher tendency to spread to adjacent organs and lymph nodes (4). Currently, radical cystectomy (RC) with pelvic lymphadenectomy is the gold standard treatment for MIBC and high-risk NMIBC. But, due to the frequent occurrence of recurrence and metastasis, patients with MIBC still exhibit unsatisfactory 5-year survival rates after receiving RC (5). There is a wide range of factors that affect the oncologic prognosis in BCa such as surgery age, gender, pathological classification, and histologic grade (6). Even if patients contain a similar stage and grade of BCa, the prognosis and clinical response may vary after RC. Thus, it is mandatory to identify a marker capable of achieving accurate preoperative risk stratification in the time of individualized medicine.

By far, the prognostic models of some clinicopathologies have been commonly used to predict the prognosis of postoperative BCa patients such as the TNM staging system. However, accurate prediction of individual tumor biology remains difficult. It is necessary to explore independent indicators which combine specific BCa biomarkers into conventional clinicopathological characteristics to allow a better prediction of the prognosis (7). Emerging evidence has recently indicated that inflammation plays a crucial role in the initiation and development of multiple cancers and patients are characterized by changes in the peripheral blood cell amounts. In addition, the systemic inflammatory response, usually based on alternative peripheral blood parameters including lymphocyte, monocyte, and hemoglobin for assessment, has been reported to be associated with worse oncologic outcomes in various cancers (8). Several indicators derived from the peripheral blood have been converted to ratios, such as lymphocyte to monocyte ratio (LMR), which have been intensively investigated as useful prognostic indicators in various kinds of cancers (9). Preoperative hemoglobin levels have also been identified as predictors for oncologic outcomes (10). We analyzed these independent indicators together to apply them to optimize the prognosis prediction of BCa patients with radical cystectomy.

To our knowledge, the prognostic value of the inflammatory response biomarkers in BCa patients undergoing RC remains obscure. In this study, we created a novel prognostic marker combining preoperative hemoglobin and LMR levels, named as the systemic inflammatory response index (SIRI). Then, the prognostic value of SIRI in BCa patients undergoing radical cystectomy was initially evaluated. The relationships of SIRI with the clinicopathological parameters, overall survival, and disease-free survival were investigated. Lastly, a prognostic nomogram combining the SIRI and TNM staging system was constructed to improve the prediction of the 3- and 5-year survival rates for BCa patients after RC.

## Patients and Methods

### Patients

This retrospective study investigated the records of a total of 203 patients who underwent RC between January 2009 and October 2018 in the urology of First Affiliated Hospital of Anhui Medical University. The inclusion criteria were as follows: 1) cystoscopy and pathological examination confirmed the diagnosis of BCa; 2) patients treated with RC; and 3) older than 18. The exclusion criteria were as follows: 1) patients with a history of other malignant tumors; 2) radiotherapy or chemotherapy prior to surgery; 3) mental abnormalities; 4) missing experimental data; and 5) lack of follow-up data. Informed consent was obtained from all patients. The study was approved by the Medical Ethics Committee of First Affiliated Hospital of Anhui Medical University (SHSY -IECKY -4.0/18-68/01) and adhered to the Declaration of Helsinki.

### Clinical Variables

Clinical and pathological variables were collected as follows: age at surgery; sex; body mass index (BMI); comprehensive complication index (CCI); and primary cancer characteristics (T-stage, N-stage, M-stage, and tumor grade). The hematological data such as lymphocytes, preoperative hemoglobin, and monocytes were collected within three days before surgery through a vein and used to calculate the LMR and SIRI. The definition of LMR was calculated as the absolute lymphocyte count divided by the absolute monocyte count.

### Patient Follow-Up

In this study, each patient in this study was followed up regularly after operation. After operation, the patients were followed up until January 20, 2019 or until death every three months for the first two years and then every six months thenceforth. During each follow-up, the postoperative tumor recurrence and survival status of the patients were collected. The end point of the follow-up was the time of the last follow-up or the time of death and the date of death, and the reasons for which were registered. Survival time is defined as the time between the operation of the patient from all causes and death or the last follow-up of the patients. Overall survival (OS) was calculated from surgery to death. Disease-free survival (DFS) was calculated from surgery to disease relapse or until the date of the last follow-up.

### Statistical Analysis

Medcalc software was used to determine the optimal cut-off level for the LMR and hemoglobin, and patients were assigned to high and low LMR groups, and high low hemoglobin groups. SIRI was established based on the combination of different preoperative hemoglobin and LMR levels. SIRI was defined as previously reported (10). Both elevated hemoglobin and elevated LMR (>127gL^-1^ and >1.98, respectively) patients were portioned to grade 1; either elevated hemoglobin or elevated LMR patients to grade 2; and both decreased hemoglobin and decreased LMR patients to grade 3.

Kaplan-Meier survival analysis was used to compare the overall survival curves (OS) and disease-free survival curves (DFS), and log-rank test was applied. The receiver operating curve (ROC) analysis was conducted for the evaluation of the LMR, hemoglobin, and SIRI, and the area under the curve (AUC) was measured and compared. Univariate and multivariate cox proportional risk regression models were conducted for the determination of the univariate and multivariate survival analyses and were used to calculate the associated hazard ratio (HR) and 95% confidence interval (CI).

According to the results from the multivariate cox analysis, the R 3.2.1 (Institute for Statistics and Mathematics, Vienna, Austria) software was used to generate the OS and DFS nomogram for the 3- and 5- year survival. The decision curve analysis (DCA) and ROC curves were applied in evaluating the predictive performance of the nomogram. These evaluations were verified internally and externally through a bootstrap with 1,000 resamples and a ten-fold cross-validation. Then, the accuracy of the nomogram was evaluated by applying a calibration curve. In the calibration curve, if the predicted value is equal to the actual observed value, the curve will fall on the ideal 45^◦^slant (11). All statistical analyses of this study were performed using the IBM SPSS 20.0 software (IBM, USA) and Graphpad Prism8 software (GraphPad Software Inc., La Jolla, CA, USA). The result was considered as statistically significant with a P-value less than 0.05.

## Results

### Patient Characteristics

This study included a total of 203 patients with BCa who underwent RC. As mentioned above, the cut-point of hemoglobin was 127 gL^-1^, and the optimal cut-off level for LMR was 1.98. The ROC curve was shown in Supporting Data **Figure S1**. As shown in **Table 1**, the baseline clinical and pathological characteristics of the patients based on the LMR and hemoglobin are summarized. After stratification, 113 (55.7%) patients were grouped into high hemoglobin. The results revealed that higher hemoglobin patients were older than lower hemoglobin patients (P*** ***= 0.016). In addition, patients with a higher hemoglobin were significantly correlated with a lower BMI level, more female patients, higher T-stage, and higher N-stage than those with a decreased hemoglobin (all P*** ***< 0.05).

**Table 1:** Clinical characteristics of the patients according to the LMR and hemoglobin.

Characteristics	All	LMR			Hemoglobin		
	patients	≤1.98	>1.98	P-value	≤127	>127	P-value
		N = 166	N = 37		N = 90	N = 113	
		Median (IQR)	Median (IQR)		Median (IQR)	Median (IQR)	
Age, years				0.077			0.016
≤65	98 (48.3)	85 (51.2)	13 (35.1)		52 (57.8)	46 (40.7)	
>65	105 (51.7)	81 (48.8)	24 (64.9)		38 (42.2)	67 (59.3)	
Sex				0.966			0.004
Male	176 (86.7)	144 (86.7)	32 (86.5)		85 (94.4)	91 (80.5)	
Female	27 (13.3)	22 (13.3)	5 (13.5)		5 (5.6)	22 (19.5)	
BMI, kg/m^2^				0.067			0.013
≤24	121 (59.6)	94 (56.6)	27 (73.0)		45 (50.0)	76 (67.3)	
>24	82 (40.4)	72 (43.4)	10 (27.0)		45 (50.0)	37 (32.7)	
CCI				0.103			0.941
≤2	128 (63.1)	109 (65.7)	19 (51.4)		57 (63.3)	71 (62.8)	
>2	75 (36.9)	57 (34.3)	18 (48.6)		33 (36.7)	42 (37.2)	
T-stage				0.406			<0.001
T1	80 (39.4)	68 (41.0)	12 (32.4)		49 (54.4)	31 (27.4)	
T2	43 (21.2)	37 (22.3)	6 (16.2)		19 (21.1)	24 (21.2)	
T3	41 (20.2)	32 (19.3)	9 (24.3)		11 (12.2)	30 (26.5)	
T4	39 (19.2)	29 (17.5)	10 (27.0)		11 (12.2)	28 (24.8)	
N-stage				0.435			0.039
N0	168 (82.8)	139 (83.7)	29 (78.4)		80 (88.8)	88 (77.9)	
N+	35 (17.2)	27 (16.3)	8 (21.6)		10 (11.1)	25 (22.1)	
M-stage				0.572			0.172
M0	194 (95.6)	158 (95.2)	36 (97.3)		88 (97.8)	106 (93.8)	
M1	9 (4.4)	8 (4.8)	1 (2.7)		2 (2.2)	7 (6.2)	
Grade				0.360			0.108
Low grade	12 (5.9)	11 (6.6)	1 (2.7)		8 (8.9)	4 (3.5)	
High grade	191 (94.1)	155 (93.4)	36 (97.3)		82 (91.1)	109 (96.5)	

Percentages may not total to 100 because of rounding.

IQR, Interquartile range; BMI, Body mass index; CCI, Comprehensive complication index; LMR, lymphocyte to monocyte ratio.

The clinicopathological characteristics of the patients according to the level of the SIRI are described in **Table 2**. According to the classification of the SIRI, 81 patients [No. (%), 39.9] were assigned to SIRI grade 1, 94 patients [No. (%): 46.3] to SIRI grade 2, and the remaining 28 patients [No. (%), 13.8] to SIRI grade 3. The results revealed that patients with a higher SIRI grade were more likely to have a lower BMI level, higher T-stage, and older compared with those with a lower SIRI grade (all P*** ***< 0.05).

**Table 2:** Clinical characteristics of the patients according to the SIRI.

Characteristic	SIRI			P-value
	Grade 1	Grade 2	Grade 3	
	No. (%)	No. (%)	No. (%)	
All patients	81 (39.9)	94 (46.3)	28 (13.8)	
Age, y				0.022
≤65	48 (59.3)	41 (43.6)	9 (32.1)	
>65	33 (40.7)	53 (56.4)	19 (67.9)	
Sex				0.051
Male	76 (93.8)	77 (81.9)	23 (82.1)	
Female	5 (6.2)	17 (18.1)	5 (17.9)	
BMI, kg/m^2^				0.018
≤24	40 (49.4)	59 (62.8)	22 (78.6)	
>24	41 (50.6)	35 (37.2)	6 (21.4)	
CCI				0.296
≤2	52 (64.2)	62 (66.0)	14 (50.0)	
>2	29 (35.8)	32 (34.0)	14 (50.0)	
T-stage				0.013
T1	43 (53.1)	31 (33.0)	6 (21.4)	
T2	18 (22.2)	20 (21.3)	5 (17.9)	
T3	10 (12.3)	23 (24.5)	8 (28.6)	
T4	10 (12.3)	20 (21.3)	9 (32.1)	
N-stage				0.140
N0	71 (87.7)	77 (81.9)	20 (71.4)	
N+	10 (12.3)	17 (18.1)	8 (28.6)	
M-stage				0.443
M0	79 (97.5)	88 (93.6)	27 (96.4)	
M1	2 (2.5)	6 (6.4)	1 (3.6)	
Grade				0.234
Low grade	7 (8.6)	5 (5.3)	0 (0.0)	
High grade	74 (91.4)	89 (94.7)	28 (100.0)	

Continuous data are presented as the mean ± standard deviation and categorical data as n (%). SIRI, systemic inflammatory response index.

For categorical variables, P-values were analyzed by chi-square tests. For continuous variables, the t-test for slope was used in generalized linear models.

### Impact of Different Clinical Factors on the OS and DFS

**Figure 1:**
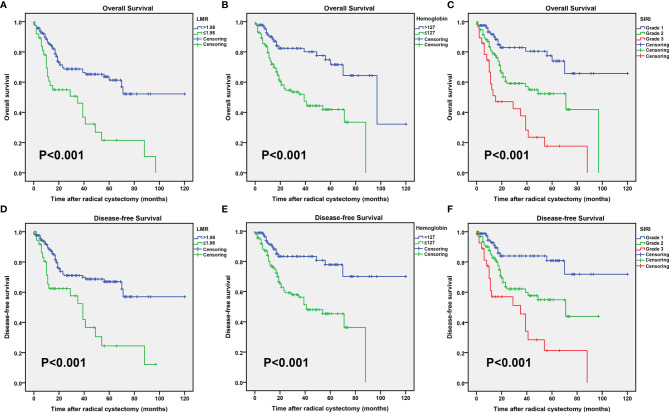
Kaplan–Meier curves for the overall survival (OS) probability in patients with radical cystectomy stratified based on the lymphocyte to monocyte ratio (LMR) **(A)**, preoperative hemoglobin **(B)**, and systemic inflammatory response index (SIRI) **(C)**. Kaplan–Meier curves for the disease-free survival (DFS) probability in patients with radical cystectomy stratified based on the LMR **(D)**, preoperative hemoglobin **(E)**, and SIRI **(F)**.

The Kaplan-Meier survival curves in the complete data set are shown in **Figure 1**. Then Kaplan-Meier survival analysis revealed that patients with increased LMR (>1.98) and hemoglobin (>127 gL^−1^) levels were both significantly associated with a better OS (P < 0.001 for both; **Figures 1A, B****)** and DFS (P < 0.001 for both; **Figures 1D, E****)**. Patients with different clinical prognoses were distinguished through combining the hemoglobin with the LMR value, we further set three subgroups. The Kaplan-Meier survival curves revealed that patients with high grade had poor OS and DFS (P < 0.001 for both; **Figures 1C, F**).

**Figure 2:**
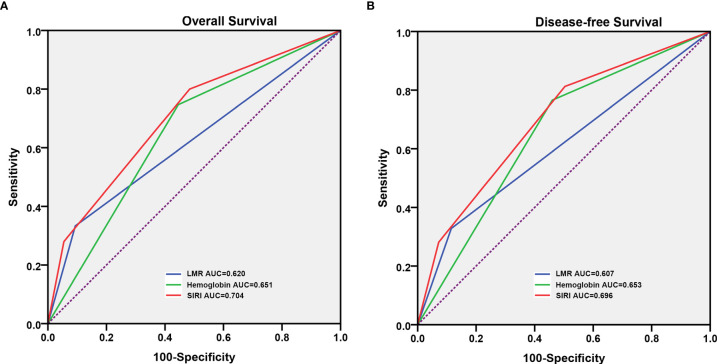
Predictive ability of the systemic inflammatory response index (SIRI) in patients who underwent radical cystectomy was compared with the lymphocyte to monocyte ratio (LMR) and hemoglobin by the receiver operating characteristic (ROC) curves in the overall survival (OS) **(A)** and disease-free survival (DFS) **(B)**.

The prognostic value of the above factors was further analyzed by comparing the ROC analysis. As indicated by the ROC analysis, the area under the curve (AUC) of the SIRI was 0.620 and 0.607, respectively, which was larger than the LMR and hemoglobin values regardless of the OS and DFS (**Figure 2**). This demonstrated that the prognostic value of the SIRI is “superior to” that of the LMR and hemoglobin. Moreover, the SIRI could better predict the OS [area under the curve (AUC): 0.704; 95% CI: 0.629–0.778] and DFS (AUC: 0.696; 95% CI: 0.618–0.774) of the patients than the LMR and hemoglobin (**Table 3**).

**Table 3:** Analysis of the predictive accuracy through the evaluation of the area under the curve (AUC).

	OS			DFS		
	AUC	95% CI	P-value	AUC	95% CI	P-value
**SIRI**	0.704	0.629–0.778		0.696	0.618–0.774	
**LMR**	0.620	0.537–0.703	0.003	0.607	0.519–0.694	0.002
Hemoglobin	0.651	0.573–0.728	0.007	0.653	0.573–0.732	0.039

P-value < 0.05 are shown in bold.

AUC, Area under the curve; OS, Overall survival; DFS, Disease-free survival; CI, confidence interval; LMR, lymphocyte to monocyte ratio; SIRI, systemic inflammatory response index.

### Univariate Analysis and Multivariate Analyses of Clinical Factors

Univariate analysis and multivariate analyses were used to evaluate the associations of the variables and overall survival (OS) and disease-free survival (DFS). As indicated in **Table 4**, the univariate analysis revealed that higher T stage, N+ stage, M1 stage, LMR > 1.98, hemoglobin > 127 gL^-1^, and higher SIRI grade were associated with a poor prognosis in patients, and the other variables had no statistical difference. Then, the critical parameters from the univariate analysis were included to evaluate the correlation with the OS through a multivariate analysis. The results identified that taking the N0, M0, and SIRI grade1 as references, the odds ratio for N+ was 2.73(95% CI, 1.62–4.60; P < 0.001), 3.31(95% CI, 1.28–8.57; P = 0.014) for M1, and 2.54(95% CI, 1.39–4.64; P = 0.002) for SIRI grade 2 was, and 4.79(95% CI, 2.41–9.50; P < 0.001) for SIRI grade 3. Similarly, the factors associated with the DFS were analyzed (**Table 5**). The univariate analysis indicated that the T-stage, N-stage, M-stage, LMR, hemoglobin, and SIRI were the factors associated with the DFS. In addition, in the multivariate analysis, the TNM stage and SIRI were independent risk predictors for the DFS. In the same way, taking the T1, N0, M0, and SIRI grade 1 as references, the odds ratio for T2 was 3.75(95% CI, 1.56–9.00; P = 0.003), 4.18(95% CI, 1.79–9.78; P = 0.001) for T3, 4.36(95% CI, 1.69–11.20; P = 0.002) for T4, 2.12(95% CI, 1.14–3.92; P = 0.017) for N+, 3.65 (95% CI, 1.36–9.79; P = 0.010) for M1, 2.19(95% CI, 1.12–4.31; P = 0.023) for SIRI grade 2, and 3.36(95% CI, 1.53–7.35; P = 0.002) for SIRI grade 3.

**Table 4:** Univariate and multivariate analyses of factors associated with the OS.

Characteristics	Univariate analyses		Multivariate analyses	
	Odds Ratio (95% CI)	P-value	Odds Ratio (95% CI)	P-value
Age, y				
≤65	Reference		Reference	
>65	1.53 (0.97–2.44)	0.070	–	0.176
Sex				
Male	Reference		Reference	
Female	0.78 (0.48–1.26)	0.312	–	0.748
BMI, kg/m^2^				
≤24	Reference		Reference	
>24	1.01 (0.52–1.96)	0.989	–	0.837
CCI				
≤2	Reference		Reference	
>2	0.93 (0.59–1.49)	0.767	–	0.366
T-stage				
T1	Reference		Reference	
T2	2.28 (1.11–4.68)	0.025	–	0.403
T3	3.82 (1.97–7.41)	<0.001	–	0.243
T4	5.56 (2.86–10.81)	<0.001	–	0.355
N-stage				
N0	Reference		Reference	
N+	3.42 (2.07–5.66)	<0.001	2.73 (1.62–4.60)	<0.001
M-stage				
M0	Reference		Reference	
M1	2.91 (1.17–7.24)	0.020	3.31 (1.28–8.57)	0.014
Grade				
Low grade	Reference		Reference	
High grade	1.90 (0.68–5.28)	0.221	–	0.506
LMR				
≤1.98	Reference		Reference	
>1.98	2.97 (1.78–4.93)	<0.001	–	0.959
Hemoglobin				
≤127	Reference		Reference	
>127	1.77 (1.11–2.82)	0.016	–	0.959
SIRI				
Grade 1	Reference		Reference	
Grade 2	2.51 (1.38–4.58)	0.003	2.54 (1.39–4.64)	0.002
Grade 3	\5.59 (2.87–10.89)	<0.001	4.79 (2.41–9.50)	<0.001

OS, Overall survival; CI, confidence interval; BMI, Body mass index; CCI, Comprehensive complication index; LMR, lymphocyte to monocyte ratio; SIRI, systemic inflammatory response index.

**Table 5:** Univariate and multivariate analyses of factors associated with DFS.

	Univariate analyses		Multivariate analyses	
Characteristics	Odds Ratio (95% CI)	P-value	Odds Ratio (95% CI)	P-value
Age, y				
≤65	Reference		Reference	
>65	1.51 (0.91–2.49)	0.108	–	0.275
Sex				
Male	Reference		Reference	
Female	0.65 (0.38–1.11)	0.114	–	0.761
BMI, kg/m^2^				
≤24	Reference		Reference	
>24	0.92 (0.44–1.93)	0.821	–	0.562
CCI				
≤2	Reference		Reference	
>2	0.83 (0.50–1.39)	0.481	–	0.197
T-stage				
T1	Reference		Reference	
T2	4.12 (1.72–9.85)	0.001	3.75 (1.56–9.00)	0.003
T3	6.73 (2.97–15.25)	<0.001	4.18 (1.79–9.78)	0.001
T4	9.59 (4.20–21.92)	<0.001	4.36 (1.69–11.20)	0.002
N-stage				
N0	Reference		Reference	
N+	3.79 (2.22–6.45)	<0.001	2.12 (1.14–3.92)	0.017
M-stage				
M0	Reference		Reference	
M1	3.55 (1.42–8.90)	0.007	3.65 (1.36–9.79)	0.010
Grade				
Low grade	Reference		Reference	
High grade	2.02 (0.63–6.49)	0.236	–	0.736
LMR				
≤1.98	Reference		Reference	
>1.98	2.92 (1.69–5.06)	<0.001	–	0.414
Hemoglobin				
≤127	Reference		Reference	
>127	1.80 (1.09–2.96)	0.021	–	0.414
SIRI				
Grade 1	Reference		Reference	
Grade 2	2.70 (1.40–5.24)	0.003	2.19 (1.12–4.31)	0.023
Grade 3	5.87 (2.82–12.22)	<0.001	3.36 (1.53–7.35)	0.002

DFS, Disease-free survival; CI, confidence interval; BMI, Body mass index; CCI, Comprehensive complication index; LMR, lymphocyte to monocyte ratio; SIRI, systemic inflammatory response index.

### Construction of a Nomogram and Validation of the Prognostic Efficiency

**Figure 3:**
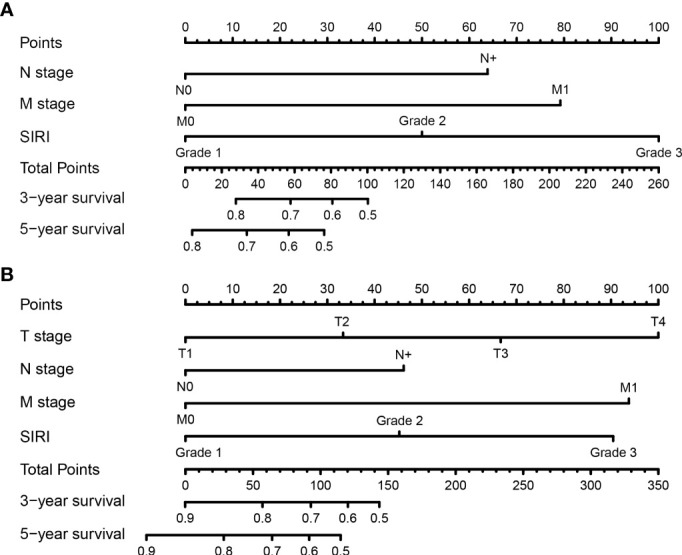
Establishment of nomograms for the prediction of the 3- and 5- year overall survival (OS) **(A)** and disease-free survival (DFS) **(B)** of patients after radical cystectomy.

All the significant independent indicators, such as the N-stage, M-stage, and SIRI, were included in the prognostic nomogram to quantitatively predict the OS of BCa patients after radical cystectomy (**Figure 3A**). Similarly, the independent indicators including the N-stage, M-stage, and SIRI were used to establish a prognostic nomogram to predict the DFS of BCa patients after RC (**Figure 3B**). The survival probability for BCa patients who underwent RC within three or five years can be predicted with the nomogram. A point was assigned to each predictor, and higher total points indicate an inferior outcome in the nomogram.

**Figure 4:**
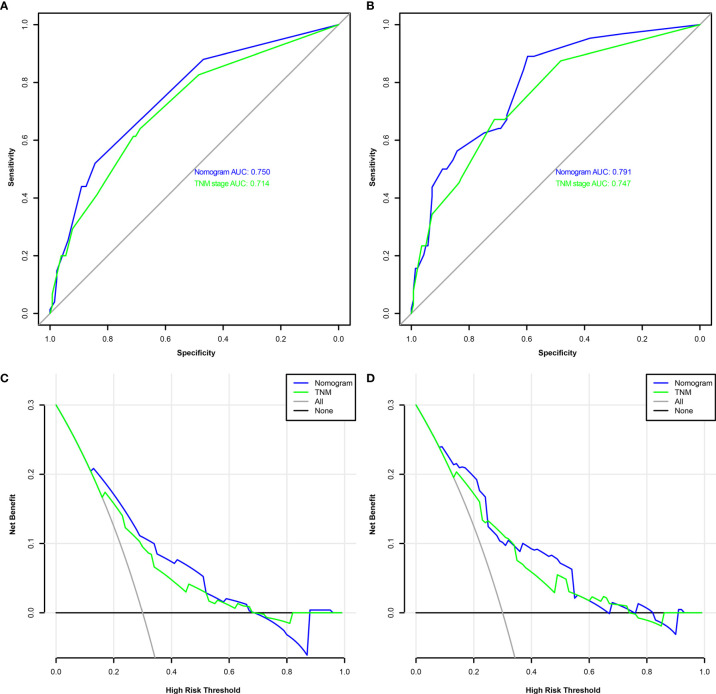
The receiver operating characteristic (ROC) analysis of the nomogram and TNM stage to predict the overall survival (OS) of the patients who underwent radical cystectomy **(A)**. The ROC analysis of the nomogram and TNM stage to the disease-free survival (DFS) of the patients who underwent radical cystectomy **(B)**. The decision curve analysis of the nomogram and TNM stage for the survival benefit in the OS **(C)**. The decision curve analysis of the nomogram and TNM stage for the survival benefit in the DFS **(D)**.

**Figure 5:**
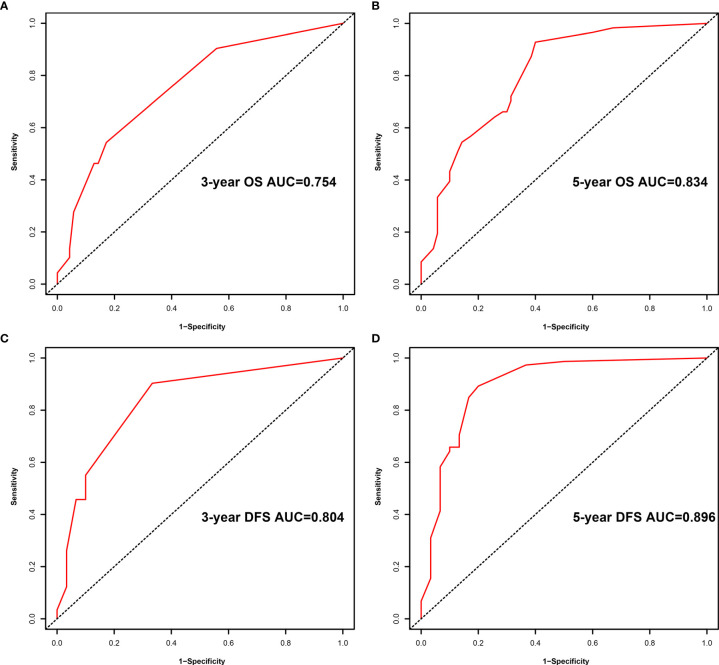
Receiver operating characteristic (ROC) analysis of the prognostic accuracy of nomogram for the 3- year overall survival (OS) **(A)**, 5- year OS **(B)**, 3- year disease-free survival (DFS) **(C)**, and 5- year DFS **(D)**.

**Figure 6:**
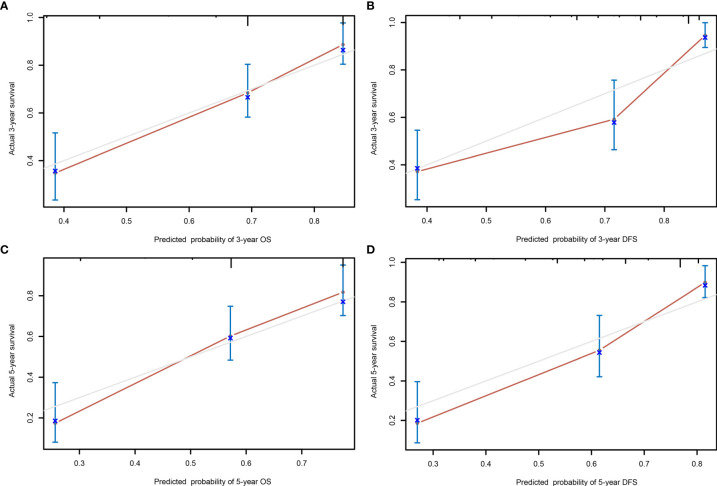
The calibration curves of the nomogram predicting the 3- year overall survival (OS) **(A)**, 5- year OS **(C)**, 3- year disease-free survival (DFS) **(B)**, and 5- year DFS **(D)**.

A comparison of the clinical benefit of the nomogram with those of the TNM criteria-based tumor staging was performed. We developed the ROC and DCA curves of these nomograms to verify that the nomograms had been well-calibrated. The AUCs of the nomogram were 0.75 and 0.791, in the analysis of the 3- and 5-year survival rates of the OS and DFS, respectively, both of which were higher than those of 0.714 and 0.747 for the TNM stage, indicating that this nomogram was more accurate in predicting the prognosis of patients with BCa after RC compared with the traditional TNM stage (**Figures 4A, B****)**. With the DCA curves, the nomograms were demonstrated to better predict the 3- and 5-year survival rates of the OS and DFS, as it added more net benefits compared with the TNM stage (**Figures 4C, D****)**. Then, in the ROC analysis of the 3- and 5-year OS and DFS, the 3- and 5-year OS AUCs of the nomogram were 0.754 and 0.834, respectively, and the 3- and 5-year DFS AUCs of the nomogram were 0.804 and 0.896, respectively, indicating that the nomogram is powerful to predict the 3- and 5-year OS and DFS (**Figure 5**). The calibration curves of these nomograms were developed, which revealed that the 3- and 5-year OS and DFS predicted by the nomograms were highly consistent with the actual observation results, indicating that the nomograms were well-calibrated (**Figure 6**).

## Discussion

In this study, we investigated the clinicopathological characteristics and prognosis of 203 BCa patients after RC. Hemoglobin and LMR were found to be independent prognostic factors that adversely predicted the overall survival and disease-free survival of BCa patients. In addition, a novel prognostic indicator named SIRI was created based on dichotomous hemoglobin and LMR. We confirmed that a high SIRI grade was significantly associated with poor clinical characteristics and high T-stage. Therefore, SIRI serves as a more objective and relatively available marker that could improve the predictive accuracy. The present study attempted to generate a nomogram to predict the OS and DFS of BCa patients after RC within three and five years based on the N-stage, M-stage, and SIRI and the N-stage, M-stage, and SIRI, respectively. Calibration plots of the nomogram predicting the 3- and 5-year OS and DFS worked well with the constructed model. Hence, as a significant predictor of the prognosis of BCa patients after RC, SIRI can be applied in risk stratification and clinical decision-making.

Recently, there is increasing evidence showing that the outgrowth, deterioration, and metastasis of cancer might be affected by systemic inflammation, thereby contributing to survival patterns changing (12). Inflammatory processes, as one of the hallmarks of cancer, involve cytokines, small inflammatory proteins, and immune cells (13). Moreover, inflammation caused by tumors can result in hematologic changes, such as lymphocytes, monocytes, and hemoglobin (14). Besides the traditional TNM stage and histologic classification, several inflammatory biomarkers and hematological-based indices, such as LMR and hemoglobin, have been reported in the prognostic evaluation of BCa (15). However, in patients with BCa undergoing RC, a combination of these frequently reported hematological and laboratory markers remains of an insignificant prognostic value.

SIRI, a combination of the hemoglobin counts and LMR, is significantly correlated with the postoperative recurrence and metastasis in patients with pancreatic cancer (16), clear cell renal cell carcinoma (17), and esophageal squamous cell carcinoma (18). The function of hemoglobin, lymphocytes, and monocytes can explain why SIRI can be considered as the prognostic predictors. Because of chronic blood loss, functional iron deficiency, and inflammation imbalance in terms of an increased expression of interleukin and tumor necrosis factor, the malignancy induces a low hemoglobin count, which is a common complication for cancer patients (19). Jung et al. (20) reported that preoperative anemic BCa patients undergoing RC had worse oncological outcomes than non-anemic bladder cancer patients. Furthermore, anemia presumably led to tumor hypoxia, thereby reducing the quality of life and treatment delivery for the patients (21). Thus, the anemia was significantly associated with disease recurrence and cancer-specific mortality for BCa patients.

LMR is an optimal excellent prognostic marker of cancer, because the high level of monocyte count represents a high tumor burden, and lymphopenia is a marker of a weak immune response. Lymphocytes are an important component of the immune system with functions responsible for immune surveillance (22). Lymphocytes can promote apoptosis and inhibit tumor growth by cytotoxicity to kill tumor cells. In general, the reduction of lymphocytes will contribute to immune disorders and impair the immunologic reaction to the tumor (23). Patients with an invasive BCa showed significantly lower lymphocyte counts than those with a superficial BCa. In most tumors, tumor cells induce circulating monocytes to differentiate into tumor-activated macrophages (TAMs) which can promote tumor proliferation, invasion, and migration by secreting cytokines and chemokines and inducing the apoptosis of activated CD8+ T cells with anti-cancer activity (24). Moreover, the density of TAMs can affect tumor angiogenesis, which leads to a poor prognosis (25). Lymphocyte and monocyte counts, as a part of the preoperative testing, are low examination cost, readily available, and can be measured repeatedly. Therefore, SIRI based on conventional laboratory tests of the hemoglobin level, lymphocyte, and monocytes counts can be used to better evaluate the prognosis of BCa patients undergoing RC. These findings may help urologists to make better decisions in terms of the clinical process and identify patients qualified for aggressive therapy.

Multiparametric magnetic resonance imaging (mpMRI) for BCa is able to accurately provide a high tissue contrast resolution, and can finely differentiate bladder wall layers (26). The Vesical Imaging-Reporting and Data System (VI-RADS) scoring system has been validated to reproducible imaging and reporting by a consensus-driven approach (27). Many recent evidences suggest that VI-RADS was a novel imaging tool that can accurately discriminate between NMIBC and MIBC patients before transurethral resection of bladder tumor (TURBT) (28). It is equally capable of being used for patient stratification for treatment planning, disease monitoring, and assessing tumor aggressiveness and response to treatment. In addition, there is considerable evidence that other hematochemical and liquid biopsy biomarkers have a significant value in predicting patient prognosis at different stages of BCA. For instance, Absolute basophil count have a predictive value for time of recurrence in patients with high-grade T1 BCa receiving bacillus Calmette–Guérin (BCG) after transurethral resection of the bladder tumor (29). Similarly, a history of type 2 diabetes mellitus is associated with worse outcomes in patients with high-grade T1 BCa (30). In addition, super-high-risk NMIBC patients with circulating tumor cells (CTCs) positive are at a high risk of local recurrence and disease progression (31). In the future, SIRI can be combined with the predictors above to more accurately and scientifically predict the prognosis of BCA patients after RC.

In recent years, increasing studies revealed that nomograms as an extremely effective prediction model can predict the survival of cancer patients (32). In this study, the results of the ROC and DCA indicated that nomograms have a better predictive performance compared to the TNM-stage. Nevertheless, the present study has the following limitations. (1) The present study was a retrospective single-center study, with a small population size of 203 patients, prone to selection bias, and there was no external validation to further verify the result. (2) Although we have tried our best to control potential confounders, we could not control the effect of other possible comorbidities or drugs, which could influence the biomarkers values. (3) The treatment of patients after RC may exist certain heterogeneity, which will lead to different clinical prognoses.

In general, SIRI, as a non-invasive, easily assessed, and repeatable prognostic index, which relied on pretreatment hemoglobin and LMR, has a prognostic value for BCa patients treated with radical cystectomy. SIRI should be combined with other factors affecting prognosis to improve a comprehensive and accurate evaluation of the prognosis of patients after RC and, thereby, direct clinical diagnosis and treatment.

## Conclusion

SIRI can be a novel independent prognostic indicator and a potential marker for therapeutic response monitoring in BCa patients after RC. The nomogram integrated with SIRI can predict the survival of BCA patients undergoing RC more objectively and reliably than the traditional TNM staging system, which will help urologists choose better clinical decisions and develop rational individualized treatment regimens.

## Data Availability Statement

The raw data supporting the conclusions of this article will be made available by the authors, without undue reservation.

## Ethics Statement

The study was approved by the Medical Ethics Committee of First Affiliated Hospital of Anhui Medical University (SHSY -IECKY -4.0/18-68/01) and adhered to the Declaration of Helsinki. The patients/participants provided their written informed consent to participate in this study.

## Author Contributions

JN, KW, JX and HZ gathered the data for statistical purposes. JN, KW, JX, YZ and CL performed the statistical analyses. JbX, CT, WL and XS checked the statistical accuracy. JN and HZ performed the literature search and wrote the first draft of the manuscript. BS, CL and BP revised and edited the final version of the manuscript. BP mentorship on every part of the research. All authors contributed to the article and approved the submitted version.

## Funding

This work was funded by the National Natural Science Foundation of China (Grant No. 81870517; 32070646); Shanghai Association for Science and Technology Commission (Grant No. 19140905700).

## Conflict of Interest

The authors declare that the research was conducted in the absence of any commercial or financial relationships that could be construed as a potential conflict of interest.

## Publisher’s Note

All claims expressed in this article are solely those of the authors and do not necessarily represent those of their affiliated organizations, or those of the publisher, the editors and the reviewers. Any product that may be evaluated in this article, or claim that may be made by its manufacturer, is not guaranteed or endorsed by the publisher.

## Supplementary Material

Supplementary Figure 1 Determination of the optimal cutoff value for the lymphocyte to monocyte ratio (LMR) **(A)** and hemoglobin**(B)** based on the Receiver operating characteristic (ROC) analysis.
