# Supplementary figures and images for: Prognostic Value of the Systemic Inflammatory Response Index in Patients Undergoing Radical Cystectomy for Bladder Cancer: A Population-Based Study
Source: Front Oncol. 2021 Aug 16;11:722151. doi: 10.3389/fonc.2021.722151 (PMC8416169; doi:10.3389/fonc.2021.722151)

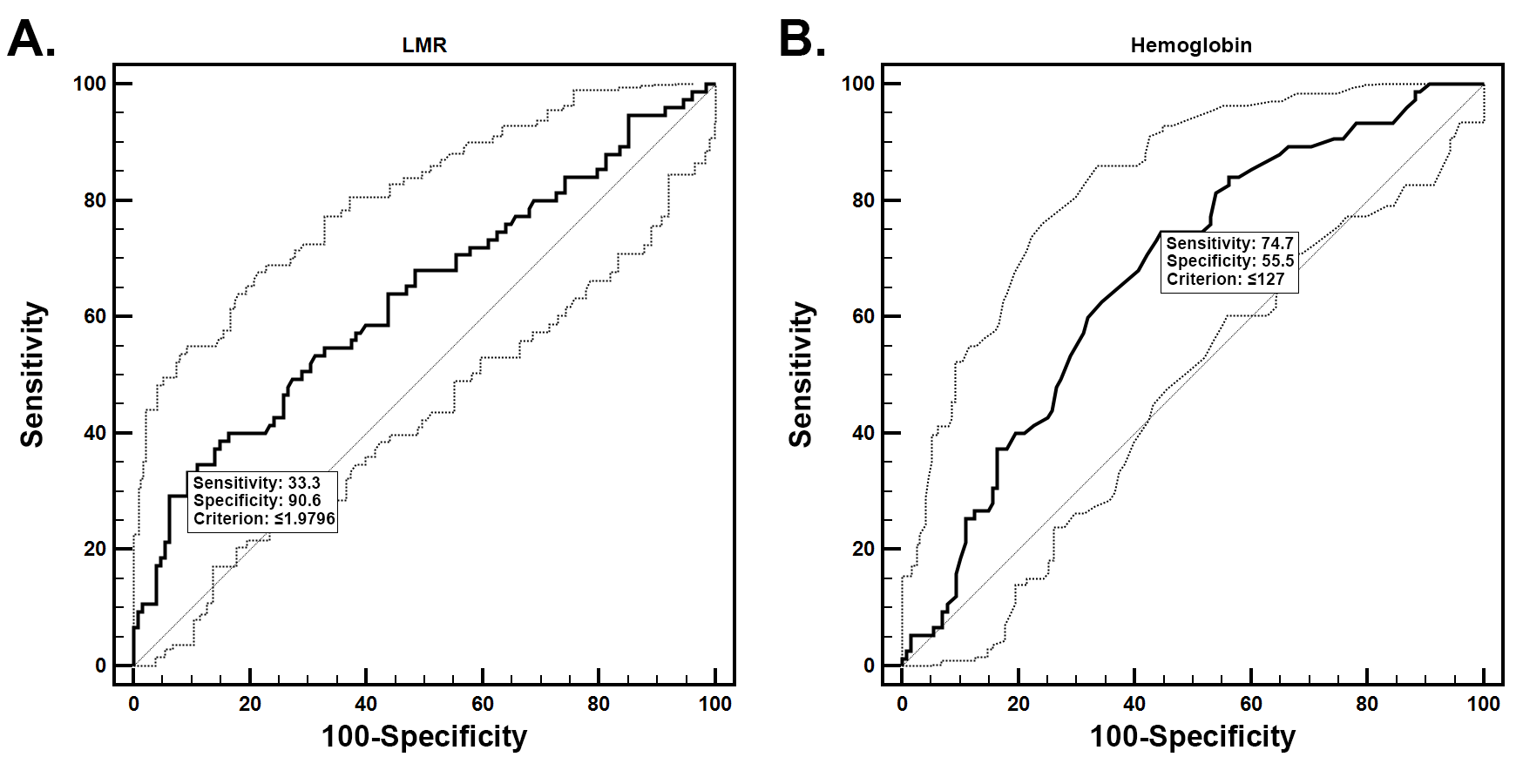

Supplement: Supplementary Figure 1 — Determination of the optimal cutoff value for the lymphocyte to monocyte ratio (LMR) (A) and hemoglobin(B) based on the Receiver operating characteristic (ROC) analysis. [file Image_1.tif]
